# High-precision force sensing using a single trapped ion

**DOI:** 10.1038/srep28078

**Published:** 2016-06-16

**Authors:** Peter A. Ivanov, Nikolay V. Vitanov, Kilian Singer

**Affiliations:** 1 Department of Physics, St. Kliment Ohridski University of Sofia, James Bourchier 5 blvd, 1164 Sofia, Bulgaria; 2 Experimentalphysik I, Universität Kassel, Heinrich-Plett-Str. 40, D-34132 Kassel, Germany

## Abstract

We introduce quantum sensing schemes for measuring very weak forces with a single trapped ion. They use the spin-motional coupling induced by the laser-ion interaction to transfer the relevant force information to the spin-degree of freedom. Therefore, the force estimation is carried out simply by observing the Ramsey-type oscillations of the ion spin states. Three quantum probes are considered, which are represented by systems obeying the Jaynes-Cummings, quantum Rabi (in 1D) and Jahn-Teller (in 2D) models. By using dynamical decoupling schemes in the Jaynes-Cummings and Jahn-Teller models, our force sensing protocols can be made robust to the spin dephasing caused by the thermal and magnetic field fluctuations. In the quantum-Rabi probe, the residual spin-phonon coupling vanishes, which makes this sensing protocol naturally robust to thermally-induced spin dephasing. We show that the proposed techniques can be used to sense the axial and transverse components of the force with a sensitivity beyond the 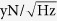 range, i.e. in the 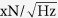 (xennonewton, 10^−27^). The Jahn-Teller protocol, in particular, can be used to implement a two-channel vector spectrum analyzer for measuring ultra-low voltages.

Over the last few years, research of mechanical systems coupled to quantum two-level systems has attracted great deal of experimental and theoretical interest^1^,^2^. Micro- and nano-mechanical oscillators can respond to very weak electric, magnetic and optical forces, which allows one to use them as highly sensitive force detectors^3^. For example, the cantilever with attonewton (10^−18^ N) force sensitivity can be used to test the violation of Newtonian gravity at sub-millimeter length scale^4^. With current quantum technologies coupling between a nanomechanical oscillator and a single spin can be achieved experimentally by using strong magnetic-field gradient. Such a coupling paves the way for sensing the magnetic force associated with the single electron spin^5^. To this end, a recent experiment demonstrated that the coherent evolution of the electronic spin of an individual nitrogen vacancy center can be used to detect the vibration of a magnetized mechanical resonator^6^.

Another promising quantum platform with application in high-precision sensing is the system of laser-cooled trapped ions, which allows excellent control over the internal and motional degrees of freedom^7^. Force sensitivity of order of 170 yN Hz^−1/2^ (10^−24^ N) was reported recently with an ensemble of ions in a Penning trap^8^. Force measurement down to 5 yN has been demonstrated experimentally using the injection-locking technique with a single trapped ion^9^. Moreover, force detection with sensitivity in the range of 1 yN Hz^−1/2^ is possible for single-ion experiments based on the measurement of the ion’s displacement amplitude^10^.

In this work, we propose ion-based sensing schemes for measuring very rapidly varying forces, which follow an earlier proposal^11^ wherein the relevant force information is mapped into the spin degrees of freedom of the single trapped ion. In contrast to^11^, the techniques proposed here do not require specific adiabatic evolution of the control parameters but rather they rely on using Ramsey-type oscillations of the ion’s spin states, which are detected via state-dependent fluorescence measurements. Moreover, we show that by using dynamical decoupling schemes, the sensing protocols become robust against dephasing of the spin states caused by thermal and magnetic-field fluctuations.

We consider a quantum system described by the Jaynes-Cummings (JC) model which can be used as a highly sensitive quantum probe for sensing of the axial force component. By applying an additional strong driving field^12^,^13^ the dephasing of the spin states induced by the residual spin-phonon interaction can be suppressed such that the sensing protocol does not require initial ground-state cooling of the ion’s vibrational state. We show that the axial force sensing can be implemented also by using a probe represented by the quantum Rabi (QR) model. Because of the absence of residual spin-motional coupling in this case, the force estimation is robust to spin dephasing induced by the thermal motion fluctuations.

Furthermore, we introduce a sensing scheme capable to extract the two-dimensional map of the applied force. Here the quantum probe is represented by the Jahn-Teller (JT) model, in which the spin states are coupled with phonons in two spatial directions. We show that the two transverse components of the force can be measured by observing simply the coherent evolution of the spin states. In order to protect the spin coherence during the force estimation we propose a dynamical decoupling sequence composed of phonon phase-shift operators, which average to zero the residual spin-phonon interaction.

We estimate the optimal force sensitivity in the presence of motional heating and find that with current ion trap technologies force sensitivity better than 1 yN Hz^−1/2^ can be achieved. Thus, a single trapped ion may serve as a high-precision sensor of very weak electric fields generated by small needle electrodes with sensitivity as low as 1 *μ*V/m Hz^−1/2^.

## 1D Force Sensing

### Jaynes-Cummings quantum probe

In our model we consider a single two-state ion with a transition frequency *ω*_0_, in a linear Paul trap with an axial trap frequency *ω*_*z*_. The interaction-free Hamiltonian is 
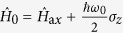
, where 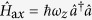 describes the small axial oscillation of the ion with 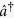 (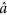) being the respective creation (annihilation) operators of phonon excitation. Consider that the ion interacts with a laser field with frequency *ω*_L_ and wave vector *k* pointing along the trap axis, which is described by the interaction Hamiltonian 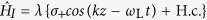 where *λ* is the respective interaction strength. We assume that the laser frequency is tuned near the red-sideband transition *ω*_L_ = (*ω*_0_ − Δ) − (*ω*_*z*_−*ω*), with detuning *ω*, while Δ is the detuning of the AC-Stark shifted states with respect to *ω*_0_. Transforming the Hamiltonian 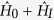 in to rotating frame by means of 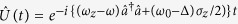 and assume rotating-wave approximation and Lamb-Dicke limit we have^14^,^15^,^16^


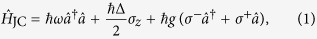


Here, *σ*_*x*,*y*,*z*_ are the Pauli matrices, *σ*^±^ are the respective raising and lowering operators for the effective spin system, *g *= *ηλ* determines the strength of the spin-phonon coupling and *η* is the Lamb-Dicke parameter (*η* ≪ 1).

The external time-varying force with a known frequency *ω*_*d*_ = *ω*_*z*_ − *ω*, e.g., *F* (*t*) = *F* cos(*ω*_*d*_*t*), displaces the motional amplitude of the ion oscillator along the axial direction, as described by the term


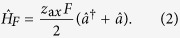


Here 
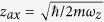
 is the spread of the zero-point wavefunction along the axial direction and *F* is the parameter we wish to estimate. The origin of the oscillating force can be a very weak electric field, an optical dipole force, spin-dependent forces created in a magnetic-field gradient or a Stark-shift gradient, etc. With the term (2) the total Hamiltonian becomes


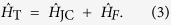


In the following, we consider the weak-coupling regime *g* ≪ *ω*, in which the phonon degree of freedom can be eliminated from the dynamics. This can be carried out by applying the canonical transformation 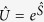 to 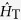 (3) such that 
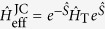
 with 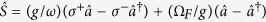
17. Keeping only the terms of order of *g*/*ω* we arrive at the following effective Hamiltonian (see the Supplement for an overview of the derivation),


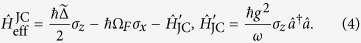


This result indicates that the spin-motional interaction in Eq. (3) shifts the effective spin frequency by the amount 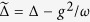, while the effect of the force term is to induce transitions between the spin states. The strength of the transition is quantified by the Rabi frequency Ω_*F*_ = *gz*_a*x*_*F*/2ħ*ω*, which is proportional to the applied force *F*. Hence the force estimation can be carried out by observing the coherent evolution of the spin population that can be read out via state-dependent fluorescence.

The last term 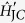 in Eq. (4) is the residual spin-motional coupling. This term affects the force estimation because it can be a source of pure spin dephasing^18^. Indeed, the *σ*_*z*_ factor in 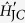 induces transitions between the eigenstates |±〉 of the operator *σ*_*x*_ depending on the vibrational state of the oscillator. As long as the oscillator is prepared initially in an incoherent vibrational state at a finite temperature this would lead to a random component in the spin energy. As we will see below, by using dynamical decoupling the effect of the pure spin dephasing can be reduced.

The sensing protocol starts by preparing the system in state 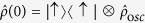, where 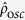 stands for the initial density operator of the oscillator. According to Eq. (4), the evolution of the system is driven by the unitary propagator 
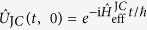
. Assuming for the moment that 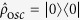 where 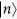 is the harmonic oscillator Fock state with *n* phonon excitations, the probability to find the system in state 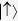 is


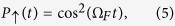


where for simplicity we set Δ = *g*^2^/*ω*, hence 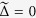. In this case, the effect of 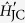 automatically vanishes such that the signal exhibits a cosine behavior according to the effective Hamiltonian (4). An initial thermal phonon distribution, however, would introduce dephasing on the spin oscillations caused by thermal fluctuations. The spin coherence can be protected, for example, by applying a sequence of fast pulses, which flip the spin states and average the residual spin-motional interaction to zero during the force estimation^19^. On the other hand, because the relevant force information is encoded in the *σ*_*x*_ term in Eq. (4), continuously applying an additional strong driving field 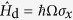 in the same basis^12^,^13^, such that 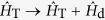, would not affect the force estimation but rather will suppress the effect of the residual spin-motional coupling. Indeed, going in the interaction frame with respect to 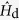, the residual spin-motional coupling becomes (see the Supplementary Information).


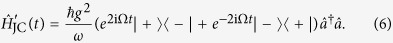


The latter result indicates that the off-resonance transitions between states |±〉 induced by 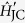 are suppressed if *g*^2^/2*ω* ≪ Ω. By separating the pulse sequences from *t* = 0 to *t*/2 with a Hamiltonian 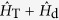, and then from *t*/2 to *t* with a Hamiltonian 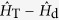, the spin states are protected from the thermal dephasing and the signal depends only on the Rabi frequency Ω_*F*_ at the final time *t*. Note that the effect of the magnetic field fluctuations of the spin states is described by an additional *σ*_*z*_ term in Eq. (4), therefore the strong driving field used here suppresses the spin dephasing caused by the magnetic-field fluctuations, as was experimentally demonstrated^20^,^21^.

**Figure 1:**
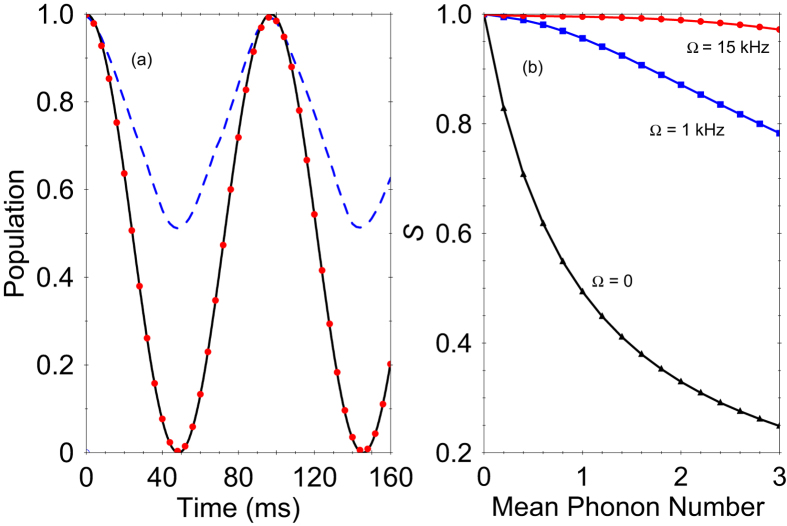
(**a**) Time-evolution of the probability to find the system in spin state 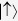 for the JC system. We compare the probabilities derived from the original Hamiltonian (3) (dots) and the effective Hamiltonian (4) (solid lines). We assume an initial thermal distribution with a mean phonon number 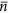 = 1.2. The parameters are set to *g* = 4 kHz, *ω* = 170 kHz, Δ = *g*^2^/2*ω*, *z*_a*x*_ = 14.5 nm, *F* = 20 yN and Ω = 10 kHz. For the same initial state but in the absence of driving field (Ω = 0), the signal loses contrast (blue dashed line). (**b**) Contrast of the Rabi oscillations defined as *S* = *P*_↑_ (*t*_2_) − *P*_↑_ (*t*_1_) with *t*_1_ = *π*/2Ω_*F*_ and *t*_2_ = *π*/Ω_*F*_ with Ω_*F*_ = 60 kHz as a function of the mean phonon number 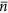.

In Fig. 1(a) we show the time evolution of the probability *P*_↑_(*t*) for an initial thermal vibrational state. Applying the driving field during the force estimation leads to reduction of the spin dephasing and hence protecting the contrast of the Rabi oscillations, see Fig. 1(b). We note that a similar technique using a strong driving carrier field for dynamical decoupling was proposed for the implementation of a high-fidelity phase gate with two trapped ions^22^,^23^.

The shot-noise-limited sensitivity for measuring Ω_*F*_ is


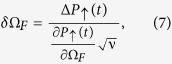


where 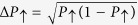 stands for the variance of the signal and ν = *T*/*τ* is the repetition number. Here *T* is the total experimental time, and the time *τ* includes the evolution time as well as the preparation and measurement times. Because our technique relies on state-projective detection, such that the preparation and measurement times are much smaller than the other time scale, we assume *τ* ≈ *t*. Using Eqs (5) and (7) we find that the sensitivity, which characterizes the minimal force difference that can be discriminated within a total experimental time of 1 s, is


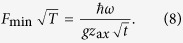


**Figure 2:**
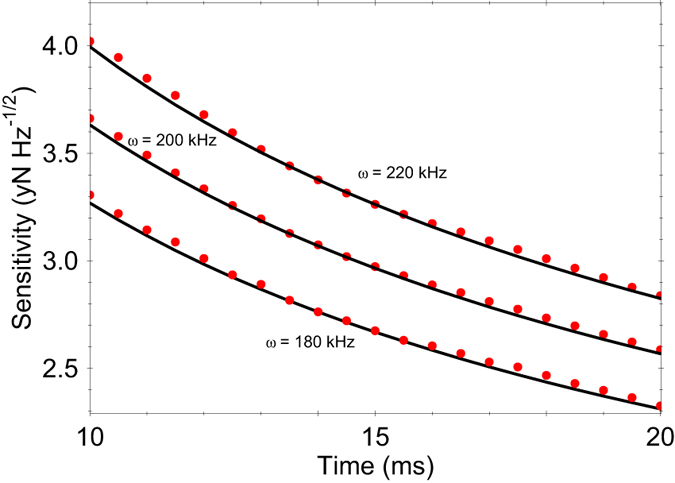
The sensitivity of the force measurement versus time t for various values of *ω*. We assume an initial thermal vibrational state with a mean phonon number 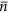 = 1. The solid lines represent the analytical result given by Eq. (8) while the dots are the exact numerical solution with the Hamiltonian (3) including the strong driving term. The other parameters are set to *g* = 4 kHz and Ω = 7 kHz.

In Fig. 2 we show the sensitivity of the force estimation versus time *t* for different frequencies *ω* assuming an initial thermal vibrational state. For an evolution time of 20 ms, force sensitivity of 2 yN Hz^−1/2^ can be achieved.

Increasing the interaction time *t* improves the force sensitivity until the random noise compromises the signal contrast. Let us now estimate the effect of the motional heating which limits the force estimation. Indeed, the heating of the ion motion causes damping of the signal, which leads to^14^


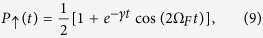


where *γ* is the decoherence rate. We assume that 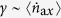 where 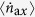 stands for the axial ion’s heating rate. Thus using Eqs (7) and (9) we find


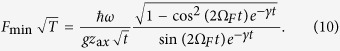


Optimizing Eq. (10) with respect to *t* and Ω_*F*_^24^ we obtain


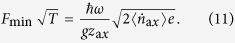


Using the parameters in Fig. 2 with *ω *= 180 kHz and assuming 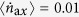 ms^−1  ^25,26 we estimate force sensitivity of 2.4 yN Hz^−1/2^. For a cryogenic ion trap with heating rate in the range of 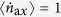 s^−1^ 10 and evolution time of *t *= 500 ms, the force sensitivity would be 0.8 yN Hz^−1/2^.

### Quantum rabi model

An alternative approach to sense the axial component of the force is to use a probe described by the quantum Rabi model,


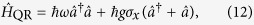


which includes the counter-rotating wave terms. This Hamiltonian can be implemented by using a bichromatic laser field along the axial direction^27^. In the weak-coupling regime, *g* ≪ *ω*, we find by using the unitary transformation 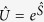 with 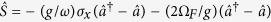 that (see the Supplement)


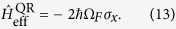


**Figure 3:**
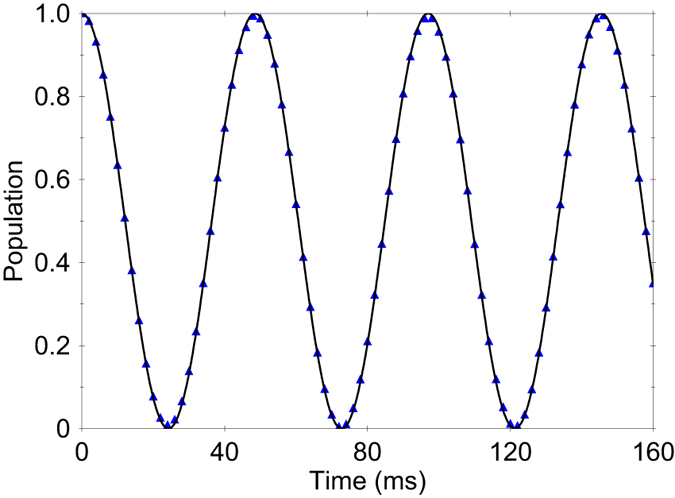
Time-evolution of the probability to find the system in spin state 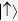 for the QR system. We assume an initial thermal vibrational state with a mean phonon number 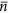 = 1.2. Due to the absence of residual spin-motion coupling the Rabi oscillations are robust with respect to the spin dephasing caused by the thermal fluctuations. We compare the probability derived from the Hamiltonian 
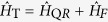
 (blue triangles) with the analytical solution *P*_↑_(t) = cos^2^(2Ω_*F*_*t*) (solod line). The parameters are set to *g* = 4 kHz, *ω *= 170 kHz, *z*_a*x*_ = 14.5 nm, *F* = 20 yN.

In contrast to Eq. (4), now the effective Hamiltonian (13) does not contain an additional residual spin-motional coupling, which implies that the spins are immune to dephasing caused by the thermal motion fluctuations, see Fig. 3. Thereby the force estimation can be carried out without additional strong driving field. Because of the extra factor of 2 in (13) now the probability to find the system in state 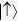 is *P*_↑_(t) = cos^2^(2Ω_*F*_*t*). Using that and following the same step as above we find that the optimal force sensitivity is,


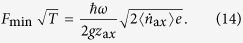


Up to now we have considered probes that are responsive only to the axial component of the force. In the following we propose a sensing technique that can be used to detect the two transverse components of the time-varying external force.

### Jahn-Teller quantum probe

In conventional ion trap sensing methods, the information on the force direction can be extracted by using the three spatial vibrational modes of the ion^10^,^28^. Such an experiment requires an independent measurement of the displacement amplitudes in each vibrational mode, which, however, increases the complexity of the measurement procedure and can lead to longer total experimental times. Here we show that by utilizing the laser-induced coupling between the spin states and the transverse ion oscillation we are able to detect the transverse components of the force by observing simply the coherent evolution of the spin states.

Indeed, let us consider the case in which the small transverse oscillations of the ion with a frequency *ω*_t_ described by the Hamiltonian 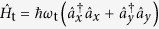 are coupled with the spin states via Jahn-Teller interaction. Such a coupling can be achieved by using bihromatic laser fields with frequencies *ω*_*b*,*r*_ = *ω*_0_ ± (*ω*_t_ − *ω*) tuned respectively near the blue- and red-sideband resonances, with a detuning *ω*, which excite the transverse *x* and *y* vibrational modes of the trapped ion. The interaction Hamiltonian of the system is given by^29^,^30^


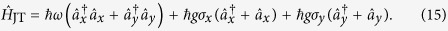


Here 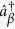 and 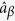 are the creation and annihilation operators of phonon excitations along the transverse direction (*β* = *x*, *y*) with an effective frequency *ω*. The last two terms in Eq. (15) describe the Jahn-Teller *E* ⊗ *e* spin-phonon interaction with a coupling strengths *g*. In the following, we assume that a classical oscillating force with a frequency *ω*_*d*_ = *ω*_t_ − *ω* displaces the vibrational amplitudes along the transverse *x* and *y* directions of the quantum oscillator described by


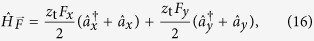


where 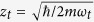 is the size of the transverse ion’s harmonic oscillator ground-state wavefunction. *F*_*x*_ and *F*_*y*_ are the two transverse components of the force we wish to estimate. With the perturbation term (16) the total Hamiltonian becomes


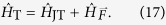


Assuming the weak-coupling regime, *g* ≪ *ω*, the two phonon modes are only virtually excited. After performing the canonical transformation 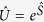 of 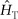 (17), where


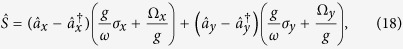


we obtain the following effective Hamiltonian (see the Supplement)


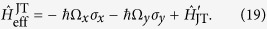


Here Ω_*x*,*y*_ = *gz*_t_*F*_*x*,*y*_/ħ*ω* are the respective driving Rabi frequencies of the transition between spin states 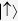 and 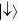. The last term in Eq. (19) is the residual spin-phonon interaction described by


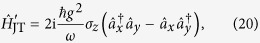


which can be a source of thermal spin dephasing as long as the two phonon modes are prepared in initial thermal vibrational states.

The two-dimensional force sensing protocol starts by preparing the system in state 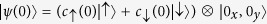, where *c*_↑,↓_ (0) are the respective initial spin probability amplitudes and 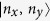 stands for the Fock state with *n*_*β*_ excitations in each phonon mode. According to the effective Hamiltonian (19) the evolution of the system is driven by the free propagator 
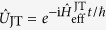
. Neglecting the residual spin-motional coupling (20) the propagator reads


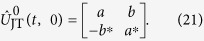


**Figure 4:**
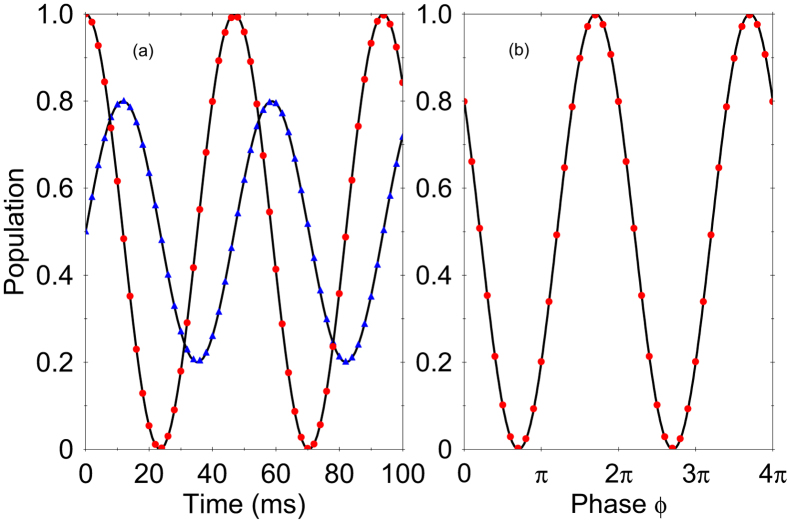
(**a**) Time-evolution of the probability to find the system in spin state 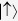 for the JT system. We compare the probability calculated from the Hamiltonian (17) assuming the initial states 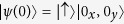 (red dots) and 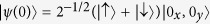 (blue triangles) with those given by the effective Hamiltonian (19) (solid lines). The parameters are set to *g *= 4 kHz, *ω *= 170 kHz, *z*_t_ = 12 nm, *F*_*x*_ = 20 yN and *F*_*y*_ = 15 yN. (**b**) Oscillations of the signal for fixed *t* as a function of the phase *ϕ* for an initial superposition spin state.

Here 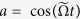 and 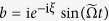 are the Cayley-Klein parameters, which depend on the rms Rabi frequency 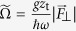, which is proportional to the magnitude of the force 
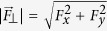
. In addition to 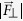, we introduce the relative amplitude parameter 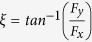. Assuming an initial state with *c*_↑_ (0) = 1, *c*_↓_ (0) = 0, the respective probability to find the system in state 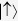 is 
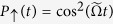
, which implies that the Rabi oscillations depends only on the magnitude of the force, see Fig. 4(a). Using Eq. (7) we find that the shot-noise-limited sensitivity for measuring the magnitude of the force is given by


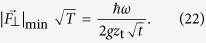


In the presence of motional heating of both vibrational modes, the signal is damped with decoherence rate 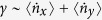, where 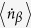 is the heating rate along the *β* spatial direction. Therefore we find that the optimal force sensitivity is


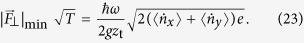


It is important that due to the strong transverse confinement the sensing scheme for measuring 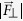 is less sensitive to the ion’s heating^31^,^32^. Using the parameters in Fig. 4 and assuming 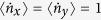 s^−1^ we estimate force sensitivity of 0.6 yN Hz^−1/2^.

In order to detect the parameter *ξ* we prepare the spin state in an initial superposition state with *c*_↑_ (0) =1/ 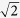 and c_↓_ (0) = *e*^i*ϕ*^/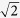. Then the probability oscillates with time as


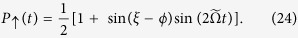


Hence, for fixed evolution time *t*, the Ramsey oscillations versus the phase *ϕ* provide a measure of the relative phase *ξ*, see Fig. 4(b).

In fact, Eq. (24) allows one to determine both the magnitude of the force 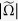 and the mixing parameter *ξ* from the same signal when plotted vs the evolution time *t*: 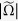 is related to the oscillation frequency and *ξ* to the oscillation amplitude. The parameter *ξ* can be determined also by varying the externally controlled superposition phase *ϕ*, until the oscillation amplitude vanishes at some value *ϕ*_*0*_; this signals the value *ξ* = *ϕ*_0_ (modulo *π*).

Finally, we discuss the dynamical decoupling schemes, which can be used to suppress the effects of the term 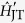 (20) during the force estimation. In that case, applying continuous driving field, e.g., along the *σ*_*x*_ direction, would reduce the thermal fluctuation induced by 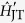, but additionally, the relevant force information, which is encoded in the *σ*_*y*_ term in (19), will be spoiled. Here we propose an alternative dynamical decoupling scheme, which follows the Carr-Purcell-Meiboom-Gill (CPMG) pulse sequence^33^,^34^, in which, however, the single instantaneous *π* pulse is replaced by the phonon phase-flip operator 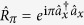. Such a phonon phase shift Δ*ω*_*x*_*τ* = *π* can be achieved by switching the RF potential of the trap by the fixed amount Δ*ω*_*x*_ for a time *τ*^35^. The effect of 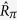 is to change the sign of the 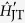 such that 
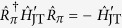
 but it leaves the other part of the Hamiltonian (19) unaffected. Using that the pulse sequence 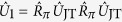 eliminates the residual spin-phonon coupling in the first order of the interaction time *t*, a high-order reduction can be achieved by the recursion 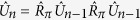, which eliminates the spin-phonon coupling up to *n*th order in *t*^36^,^37^.

### Summary and outlook

We have proposed quantum sensing protocols, which rely on mapping the relevant force information onto the spin degrees of freedom of the single trapped ion. The force sensing is carried out by observing the Ramsey-type oscillations of the spin states, which can be detected via state-dependent fluorescence. We have considered quantum probes represented by the JC and QR systems, which can be used to sense the axial component of the force. We have shown that when using a JC system as a quantum probe, one can apply dynamical decoupling schemes to suppress the effect of the spin dephasing during the force estimation. When using a QR system as a probe, the absence of a residual spin-phonon coupling makes the sensing protocol robust to thermally-induced spin dephasing. Furthermore, we have shown that the transverse-force direction can be measured by using a system described by the JT model, in which the spin states are coupled with the two spatial phonon modes. Here the information of the magnitude of the force and the relative ratio can be extracted by observing the time evolution of the respective ion’s spin states, which simplify significantly the experimental procedure.

Tuning the trap frequencies over the broad range, the force sensing methods proposed here can be employed to implement a spectrum analyzer for ultra-low voltages. Moreover, because in the force-field direction sensing the mutual ratio can be additionally estimated our method can be used to implement a two-channel vector spectrum analyzer. Finally, the realization of the proposed force sensing protocols are not restricted only to trapped ions but could be implemented with other quantum optical setups such as cavity-QED^38^ or circuit-QED systems^39^.

After the submission of the manuscript we become aware for related experimental force sensing work^40^.

## Additional Information

**How to cite this article**: Ivanov, P. A. *et al*. High-precision force sensing using a single trapped ion. *Sci. Rep.* **6**, 28078; doi: 10.1038/srep28078 (2016).

## Supplementary Material

Supplementary Information
