# Supplementary material for: High-precision force sensing using a single trapped ion
Source: Sci Rep. 2016 Jun 16;6:28078. doi: 10.1038/srep28078 (PMC4910112; doi:10.1038/srep28078)
Supplement: Supplementary Information [file srep28078-s1.pdf]

Supplemental Material for "High-precision force sensing using a single trapped ion"

Peter A. Ivanov[1], Nikolay V. Vitanov[1] and Kilian Singer[2]
[1]*Department of Physics, St. Kliment Ohridski University of Sofia, James Bourchier 5 blvd, 1164 Sofia, Bulgaria*
[2]*Experimentalphysik I, Universität Kassel, Heinrich-Plett-Str. 40, D-34132 Kassel, Germany*

ELIMINATION OF THE VIBRATIONAL DEGREE OF FREEDOM

Let us make the canonical transformation of Hamiltonian $\hat{H}=\hat{H}_0+\hat{H}_{\rm int}$,

$$\begin{aligned}\hat{H}_{\rm eff} &= e^{-\hat{S}}\hat{H}e^{\hat{S}}=\hat{H}_0+\hat{H}_{\rm int}+[\hat{H}_0,\hat{S}]+[\hat{H}_{\rm int},\hat{S}]\\ &+\tfrac{1}{2}[[\hat{H}_0,\hat{S}],\hat{S}]+\tfrac{1}{2}[[\hat{H}_{\rm int},\hat{S}],\hat{S}]+\ldots.\end{aligned} \tag{1}$$

Our goal is to choose $\hat{S}$ in a such a way that all terms of order g in $\hat{H}_{\rm eff}$ are canceled and the first term describing the spin-boson interaction is of order g^2/ω. If we determine $\hat{S}$ by the condition

$$\hat{H}_{\rm int}+[\hat{H}_0,\hat{S}]=0, \tag{2}$$

then the effective Hamiltonian becomes

$$\hat{H}_{\rm eff}\approx\hat{H}_0+\tfrac{1}{2}[\hat{H}_{\rm int},\hat{S}]. \tag{3}$$

Let us consider the time-dependent operator $\hat{S}(t)=e^{{\rm i}\hat{H}_0t/\hbar}\hat{S}e^{-{\rm i}\hat{H}_0t/\hbar}$, which obeys the Heisenberg equation ${\rm i}\hbar\dot{\hat{S}}(t)=[\hat{S}(t),\hat{H}_0]$. Using Eq. (2) we arrive at the equation

$${\rm i}\hbar\dot{\hat{S}}(t)=\hat{H}_{\rm int}(t), \tag{4}$$

where $\hat{H}_{\rm int}(t)=e^{{\rm i}\hat{H}_0t/\hbar}\hat{H}_{\rm int}e^{-{\rm i}\hat{H}_0t/\hbar}$. Solving Eq. (4) we determine the desired operator $\hat{S}$.

Jaynes-Cummings model

We identify $\hat{H}_0=\hbar\omega\hat{a}^{\dagger}\hat{a}$ and $\hat{H}_{\rm int}=\hbar g(\sigma^{-}\hat{a}^{\dagger}+\sigma^{+}\hat{a})+\frac{z_{\rm ax}F}{2}(\hat{a}^{\dagger}+\hat{a})$. Using Eq. (4) we obtain

$$\hat{S}=\frac{g}{\omega}(\sigma^{+}\hat{a}-\sigma^{-}\hat{a}^{\dagger})+\frac{z_{\rm ax}F}{2\hbar\omega}(\hat{a}-\hat{a}^{\dagger}), \tag{5}$$

which fulfills the condition (2). For the effective Hamiltonian we derive

$$\begin{aligned}\hat{H}_{\rm eff} &= \hbar\omega\hat{a}^{\dagger}\hat{a}+\hbar\left(\Delta-\frac{g^2}{2\omega}\right)\sigma_z-\hbar\Omega_F\sigma_x\\ &-\frac{\hbar g^2}{\omega}\sigma_z\hat{a}^{\dagger}\hat{a}-\frac{\hbar g^2}{2\omega}-\frac{z_{\rm ax}^2F^2}{4\hbar\omega}+\hat{H}',\end{aligned} \tag{6}$$

where $\Omega_F=gz_{\rm ax}F/2\hbar\omega$ is the Rabi frequency and $\hat{H}'=\frac{1}{3}[[\hat{H}_{\rm int},\hat{S}],\hat{S}]+\ldots$ contains the higher-order terms in (1). We find

$$\begin{aligned}\frac{1}{3}[[\hat{H}_{\rm int},\hat{S}],\hat{S}] &= \frac{2g^2z_{\rm ax}F}{3\omega^2}\sigma_z(\hat{a}^{\dagger}+\hat{a})-\frac{4\hbar g^3}{3\omega^2}(\sigma^{-}\hat{a}^{\dagger}+\sigma^{+}\hat{a})\\ &-\frac{4\hbar g^3}{3\omega^2}(\sigma^{-}\hat{a}^{\dagger}\hat{a}^{\dagger}\hat{a}+\sigma^{+}\hat{a}^{\dagger}\hat{a}\hat{a}).\end{aligned} \tag{7}$$

As long as $g/\omega\ll1$ the higher-order terms can be neglected and thus the lowest-order effective Hamiltonian is given by Eq. (6).

Quantum Rabi Model

Here the interaction Hamiltonian is $\hat{H}_{\rm int} = \hbar g\sigma_x(\hat{a}^\dagger + \hat{a}) + \frac{z_{\rm ax}F}{2}(\hat{a}^\dagger + \hat{a})$ and the canonical transformation is given by the operator

$$\hat{S} = \frac{g}{\omega}\sigma_x(\hat{a} - \hat{a}^\dagger) + \frac{z_{\rm ax}F}{2\hbar\omega}(\hat{a} - \hat{a}^\dagger). \tag{8}$$

The effective Hamiltonian is

$$\hat{H}_{\rm eff} = \hbar\omega\hat{a}^\dagger\hat{a} - 2\hbar\Omega_F\sigma_x - \frac{\hbar g^2}{\omega} - \frac{(z_{\rm ax}F)^2}{4\hbar\omega}. \tag{9}$$

Remarkably, due to the equality $[[\hat{H}_{\rm int}, \hat{S}], \hat{S}] = 0$ all higher-order terms in Eq. (1) vanish.

Jahn-Teller Model

Following the same procedure we have

$$\begin{aligned}
&\hat{H}_0 = \hbar\omega(\hat{a}_x^\dagger\hat{a}_x + \hat{a}_y^\dagger\hat{a}_y),\\
&\hat{H}_{\rm int} = \hbar g\sigma_x(\hat{a}_x^\dagger + \hat{a}_x) + \hbar g\sigma_y(\hat{a}_y + \hat{a}_y) + \frac{z_{\rm t}F_x}{2}(\hat{a}_x^\dagger + \hat{a}_x)\\
&+\frac{z_{\rm t}F_y}{2}(\hat{a}_y^\dagger + \hat{a}_y).
\end{aligned} \tag{10}$$

In this case the canonical transformation is represented by the operator

$$\begin{aligned}
\hat{S} \;=\; &\frac{g}{\omega}\sigma_x(\hat{a}_x - \hat{a}_x^\dagger) + \frac{g}{\omega}\sigma_y(\hat{a}_y - \hat{a}_y^\dagger) + \frac{z_{\rm t}F_x}{2\hbar\omega}(\hat{a}_x - \hat{a}_x^\dagger)\\
&+\frac{z_{\rm t}F_y}{2\hbar\omega}(\hat{a}_y - \hat{a}_y^\dagger).
\end{aligned} \tag{11}$$

Using Eq. (11) we obtain the following effective Hamiltonian

$$\begin{aligned}
\hat{H}_{\rm eff} \;=\; &\hbar\omega(\hat{a}_x^\dagger\hat{a}_x + \hat{a}_y^\dagger\hat{a}_y) - \hbar\Omega_x\sigma_x - \hbar\Omega_y\sigma_y - 2{\rm i}\frac{\hbar g^2}{\omega}\\
&\times\sigma_z(\hat{a}_x\hat{a}_y^\dagger - \hat{a}_x^\dagger\hat{a}_y) - \frac{2\hbar g^2}{\omega} - \frac{z_{\rm t}^2|\vec{F}_\perp|^2}{4\hbar\omega} + \hat{H}',
\end{aligned} \tag{12}$$

where $\Omega_{x,y} = gz_{\rm t}F_{x,y}/\hbar\omega$ are the respective Rabi driving frequencies. The next higher-order terms in $\hat{H}'$ (12) are given by

$$\begin{aligned}
\frac{1}{3}[[\hat{H}_{\rm int}, \hat{S}], \hat{S}] \;=\; &2{\rm i}\frac{g^2 z_{\rm t}F_x}{\omega^2}\sigma_z(\hat{a}_y^\dagger - \hat{a}_y) - 2{\rm i}\frac{g^2 z_{\rm t}F_y}{\omega^2}\sigma_z(\hat{a}_x^\dagger - \hat{a}_x)\\
&-\frac{4\hbar g^3}{\omega^2}\sigma_y\{(\hat{a}_y^\dagger + \hat{a}_y)(1 + 2\hat{n}_x) - 2\hat{a}_x^{\dagger 2}\hat{a}_y\\
&-2\hat{a}_x^2\hat{a}_y^\dagger\} - \frac{4\hbar g^3}{\omega^2}\sigma_x\{(\hat{a}_x^\dagger + \hat{a}_x)(1 + 2\hat{n}_y)\\
&-2\hat{a}_y^{\dagger 2}\hat{a}_x - 2\hat{a}_y^2\hat{a}_x^\dagger\}.
\end{aligned} \tag{13}$$

DYNAMICAL DECOUPLING

Let us consider the Jaynes-Cummings quantum probe in the presence of additional strong carrier driving field

$$\hat{H} = \hat{H}_{\rm JC} + \hat{H}_F + \hat{H}_{\rm d}, \quad \hat{H}_{\rm d} = \hbar\Omega(\sigma_+ e^{{\rm i}\phi} + \sigma_- e^{-{\rm i}\phi}). \tag{14}$$

Here Ω is the Rabi frequency for resonant carrier transition and ϕ is the respective phase where we set $\phi = 0$. The effect of the strong carrier excitation is to create dressed states defined by $\sigma_x\left|\pm\right\rangle = \pm\left|\pm\right\rangle$ which are separated by

energy gap $\hbar\Omega$. These states are immune to dephasing caused by the thermal fluctuations. Indeed, expressing the effective Hamiltonian in the dressed state basis $\sigma_x\left|\pm\right\rangle = \pm\left|\pm\right\rangle$ we have

$$\hat{H}_{\rm eff}^{\rm JC} = \hbar(\Omega - \Omega_F)(|+\rangle\langle+| - |-\rangle\langle-|) - \frac{\hbar g^2}{\omega}(|+\rangle\langle-| + |-\rangle\langle+|)\hat{a}^{\dagger}\hat{a}. \tag{15}$$

In this picture the effect of the thermally induce fluctuations is to drive transition between $|+\rangle$ and $|-\rangle$ states. However, as long as the energy gap Ω is much higher than g^2/ω ($\Omega \gg g^2/\omega$) such transitions are highly supressed which allows to neglect the second term in (15) in the rotating-wave approximation. In order to remove the dependence of the Rabi frequency Ω in the measured signal we suggest to use a spin-echo technique. First the system is prepared in the state $|\uparrow\rangle = \frac{1}{\sqrt{2}}(|+\rangle + |-\rangle)$ which evolves under the action of Hamiltonian (15) for a time period $t/2$ such that we have $|\psi(t/2)\rangle = (e^{-{\rm i}(\Omega-\Omega_F)t/2}|+\rangle + e^{{\rm i}(\Omega-\Omega_F)t/2}|-\rangle)/\sqrt{2}$. Then the phase of the strong carrier driving field is switch by π and the state evolves to $|\psi(t/2)\rangle \rightarrow |\psi(t)\rangle = (e^{{\rm i}\Omega_F t}|+\rangle + e^{-{\rm i}\Omega_F t}|-\rangle)/\sqrt{2}$. Final read-out of the ion states in the $|\uparrow\rangle$, $|\downarrow\rangle$ basis yield probability outcome $P_{\uparrow}(t) = \cos^2(\Omega_F t)$.
